# Knockdown of the Long Noncoding RNA LUCAT1 Inhibits High-Glucose-Induced Epithelial-Mesenchymal Transition through the miR-199a-5p–ZEB1 Axis in Human Renal Tubular Epithelial Cells

**DOI:** 10.1155/2020/8895003

**Published:** 2020-12-28

**Authors:** Li-Cai Zhang, Zong-Bin Wei, Shui-Fu Tang

**Affiliations:** ^1^Department of Nephropathy, The First Affiliated Hospital of Guangzhou University of Chinese Medicine, Guangzhou, 510405 Guangdong Province, China; ^2^Department of Urology, Neijiang Traditional Chinese Medicine Hospital, Neijiang, 641100 Sichuan Province, China

## Abstract

Renal fibrosis, the leading cause of end-stage renal disease and in which epithelial-mesenchymal transition (EMT) plays a central role, has a complex pathogenesis that is not fully understood. Therefore, we investigated the role of the long noncoding RNA LUCAT1 in the EMT of renal tubular epithelial cells under high-glucose (HG) conditions and the underlying mechanism involved. In this study, we established HG and normal glucose groups of HK-2 cells by treating HK-2 cells 30.0 or 5.5 mmol/L glucose, respectively. To investigate the roles of LUCAT1 and miR-199a-5p in HG-induced EMT, we transfected the HG group with negative control small interfering RNA (siRNA), siRNA targeting LUCAT1, negative control microRNA, or an miR-199a-5p mimic. The results of the quantitative reverse transcription PCR indicated that the LUCAT1 level in the HG group was increased, whereas the miR-199a-5p level was decreased. The EMT in the cells was induced by treatment with HG but was weakened by LUCAT1 knockdown or miR-199a-5p overexpression, which both also inhibited the HG-induced phosphorylation of SMAD3. Moreover, LUCAT1 and ZEB1 mRNA comprised the same microRNA response elements of miR-199a-5p. LUCAT1 knockdown had no effect on the miR-199a-5p level but decreased the HG-induced upregulation of ZEB1. In conclusion, HG conditions induced the upregulation of LUCAT1, and LUCAT1 knockdown inhibited the EMT in HG-treated HK-2 cells. LUCAT1 likely promotes HG-induced EMT through ZEB1 by sponging miR-199a-5p.

## 1. Introduction

Renal fibrosis, the leading cause of end-stage renal disease [1], has a complex pathogenesis that is not fully understood [2]. The epithelial-mesenchymal transition (EMT) plays a central role in the progression of renal fibrosis [3]. As the EMT of renal tubular epithelial cells is an early and reversible process, we speculate that its inhibition or reversal should have great significance in controlling renal fibrosis. Increasing evidence has indicated that long noncoding RNAs (lncRNAs), which are a type of ncRNAs with a transcript length of more than 200 nt [4, 5], play an important role in the pathogenesis of EMT implicated in renal fibrosis [6, 7]; however, their role has not been fully elucidated. Therefore, in-depth investigations of the functions and regulatory mechanisms of lncRNAs in EMT would aid in the development of new treatments and interventions for delaying the progression of renal fibrosis.

Diabetic nephropathy is the main cause of end-stage renal disease, and its major pathologic feature is renal fibrosis [8]. Many studies have indicated that lncRNAs participate in the pathogenesis and progression of diabetic nephropathy [9]. Various lncRNAs, including lncRNA metastasis-associated lung adenocarcinoma transcript 1 (MALAT1) and lncRNA ZEB1-AS1, are reportedly dysregulated and can regulate the EMT in renal tubular epithelial cells under a high-glucose (HG) environment [10, 11]. However, the role of other lncRNAs in HG-induced EMT has not yet been fully elucidated. lncRNA lung cancer-associated transcript 1 (LUCAT1) has a pivotal role in malignant tumors such as lung cancer, breast cancer, and gastric cancer [12–14]. Importantly, some studies have reported that LUCAT1 is dysregulated under HG conditions [15, 16]. The expression of LUCAT1 was increased in HG-treated cardiomyocytes, and its silencing could alleviate the cardiomyocyte injury and apoptosis induced by HG [16]. Moreover, the overexpression of LUCAT1 can suppress the protein expression of inducible nitric oxide synthase in lung cells under HG conditions [15]. Therefore, we hypothesized that LUCAT1 may play a regulatory role in renal tubular epithelial cells under HG conditions.

In this study, we aimed to determine the roles of LUCAT1 in the HG-induced EMT of renal tubular epithelial cells and investigate the underlying mechanisms involved.

## 2. Materials and Methods

### 2.1. Cell Culture

The proximal tubule epithelial cell line HK-2 was purchased from the Cell Bank of the Typical Culture Storage Committee of the Chinese Academy of Sciences (Shanghai, China). The cells were cultured in Dulbecco's modified Eagle's medium/F12 medium (1 : 1) containing 10% fetal bovine serum (Gibco, Carlsbad, CA, USA) at 37°C in a humidified atmosphere containing 5% CO_2_.

### 2.2. siRNA Transfection and Experimental Grouping

We designed three small interfering RNAs (siRNAs) targeting LUCAT1 (siLUCAT1) and labeled them as siLUCAT1-1, siLUCAT1-2, and siLUCAT1-3, which were then synthesized by Suzhou GenePharma Co., Ltd. (Suzhou, China). The sense sequences (5′–3′) of the three siRNAs were CCAGACCUCCAGAAACCAUTT (siLUCAT1-1), GGAACUCUUAUGGGACCUUTT (siLUCAT1-2), and CCAACUUGCUGUUUGCUAUTT (siLUCAT1-3), respectively. The negative control siRNA (siNC), negative control microRNA (miR-NC), and miR-199a-5p mimic were also purchased from Suzhou GenePharma Co., Ltd. HK-2 cells (5 × 10^5^) were seeded in 6-well plates and were transfected at ~80% confluence with the siRNAs or microRNAs using Lipofectamine RNAiMAX Transfection Reagent (Invitrogen, Carlsbad, CA, USA).

To investigate the role of LUCAT1, we divided HK-2 cells into four groups and treated them as follows: the normal glucose (NG) group, cultured in complete culture medium containing 5.5 mmol/L glucose; the HG group, cultured in complete culture medium containing 30 mmol/L glucose; the HG+siNC group, transfected with siNC and cultured in complete culture medium containing 30 mmol/L glucose; and the HG+siLUCAT1 group, transfected with siLUCAT1 and cultured in complete culture medium containing 30 mmol/L glucose. Similarly, to investigate the role of miR-199a-5p, we divided HK-2 cells into HG+miR-NC group, which we transfected with miR-NC and cultured in complete culture medium containing 30 mmol/L glucose, and HG+miR-199a-5p group, which we transfected with the miR-199a-5p mimic and cultured in complete culture medium containing 30 mmol/L glucose. The cells were transfected for 48 h and harvested (at ~1 × 10^6^ cells per well) for quantitative reverse transcription polymerase chain reaction and western blot assay. The cells were transfected for 24 h and harvested (at ~8 × 10^5^ cells per well) for Transwell cell migration assay.

### 2.3. Quantitative Reverse Transcription Polymerase Chain Reaction

The total RNA from various groups of cells (~2 × 10^6^) were isolated using TRIzol reagent. The purity and concentration of the total RNA were measured using BioPhotometer Plus (Eppendorf, Hamburg, Germany). Then, 1 *μ*g total RNA was reverse transcribed using M-MLV reverse transcriptase (Promega, Madison, WI, USA) according to the manufacturer's instructions. Thereafter, the quantitative polymerase chain reaction (qPCR) was performed using AceQ qPCR SYBR Green Master Mix (Vazyme Biotech Co., Ltd., Nanjing, China) on ABI PRISM 7300 Sequence Detection System. The reference control gene for LUCAT1 detection was *β*-actin, whereas that for miR-199a-5p detection was U6 small nuclear 1. The reverse transcription primer for LUCAT1 and *β*-actin was a random primer, whereas those for miR-199a-5p and U6 small nuclear 1 were 5′-CTCAACTGGTGTCGTGGAGTCGGCAATTCAGTTGAGGAACAGGT-3′ and 5′-AACGCTTCACGAATTTGCGT-3′, respectively. The PCR primers were as follows: LUCAT1 forward 5′-TGAGACTTAGCGTGCCTGTA-3′, reverse 5′-GGTAAGTGTAGCATCAGGACAA-3′; *β*-actin forward 5′-GCATGGGTCAGAAGGATTCCT-3′, reverse 5′-TCGTCCCAGTTGGTGACGAT-3′; miR-199a-5p forward 5′-ACACTCCAGCTGGGCCCAGTGTTCAGACTACCT-3′, reverse 5′-CTCAACTGGTGTCGTGGA-3′; and U6 small nuclear 1 forward 5′-CTCGCTTCGGCAGCACA-3′, reverse 5′-AACGCTTCACGAATTTGCGT-3′.

### 2.4. Western Blot Assay

Cells (~3 × 10^6^) from each group were harvested and lysed in RIPA lysis buffer to isolate the total protein, whose concentration was determined using a bicinchoninic acid protein assay kit (Beyotime Institute of Biotechnology, Shanghai, China). The proteins were electrophoresed in sodium dodecyl sulfate-polyacrylamide gel, and the protein bands were electrophoretically transferred onto a polyvinylidene difluoride membrane. Afterwards, the membrane was washed with 1× Tris-buffered saline containing 1‰ Tween-20 (TBST) and incubated with horizontal shaking in freshly prepared 5% skimmed milk for 1 h at 25 ± 2°C. Then, the membrane with TBST was washed and incubated with diluted primary antibody solution at 4°C overnight. On the next day, the membrane was washed three times with 1× TBST for 5 min each time and incubated with diluted secondary antibody solution for 1 h at 25 ± 2°C. Subsequently, the membrane was washed three times with 1× TBST for 5 min each time and was incubated with Immobilon Western Chemiluminescent HRP Substrate, and the band signals were exposed to an X-ray film. Image-Pro Plus software was used to quantify the integrated optical density (IOD) of each band on the X-ray film. The relative protein level was calculated using the following formula: relative protein level = IOD of the target protein/IOD of GAPDH.

### 2.5. Transwell Cell Migration Assay

After the treatment of HK-2 cells with the different glucose and transfection conditions, 1 × 10^5^ cells were resuspended in 100 *μ*L serum-free medium and added to the upper chamber of a Transwell cell culture plate, while 600 *μ*L complete medium was added to the lower chamber. Then, after 24 h of incubation in a 5% CO_2_ incubator at 37°C, the cells in the upper chamber were wiped off with a cotton swab, and the chamber was washed once with phosphate-buffered saline (PBS). Afterwards, the cells that had passed through the membrane to the lower chamber were fixed with 4% paraformaldehyde for 10 min. Thereafter, the cells were washed once with PBS, stained with crystal violet for 10 min, and washed twice with PBS. Finally, the stained cells were observed under a microscope, and images were captured using an attached camera.

### 2.6. Statistical Analysis

GraphPad Prism version 7 software (GraphPad Software, San Diego, CA, USA) was used for all the statistical analyses. The experimental data of each group are expressed as the mean ± standard deviation. The unpaired *t*-test was used to analyze the significance of differences between two groups, whereas one-way analysis of variance was used to analyze the significance of differences among more than two groups. *P* < 0.05 was considered statistically significant.

## 3. Results

### 3.1. LUCAT1 Level Is Elevated in HG-Treated HK-2 Cells

**Figure 1:**
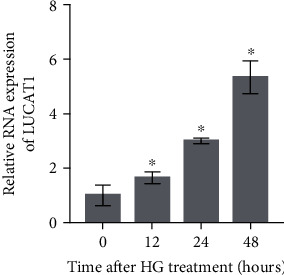
Expression levels of the long noncoding RNA LUCAT1 in high-glucose- (HG-) treated HK-2 cells measured using quantitative reverse transcription PCR. ^∗^*P* < 0.05, compared with the expression level at 0 h.

To explore the role of LUCAT1 in relation to the effects induced by HG, we measured the expression level of LUCAT1 in HG-treated HK-2 cells. As shown in Figure 1, LUCAT1 expression level increased gradually after 12, 24, and 48 h of HG treatment, suggesting that this lncRNA may play a role in regulating the damage caused by HG.

### 3.2. LUCAT1 Knockdown Inhibits the EMT of HG-Treated HK-2 Cells

**Figure 2:**
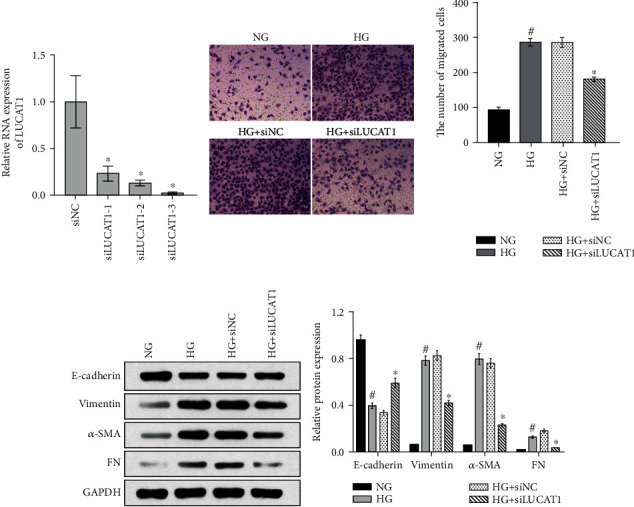
Knockdown of LUCAT1 inhibits the epithelial-mesenchymal transition (EMT) of high-glucose- (HG-) treated HK-2 cells. (a) LUCAT1 level in HK-2 cells transfected with negative control small interfering RNA (siRNA) (siNC) or three siRNAs targeting LUCAT1 (siLUCAT1; labeled as siLUCAT1-1, siLUCAT1-2, and siLUCAT1-3, respectively). (b, c) Migration capability (b) and expression levels of EMT-related proteins (c) of cells in the normal glucose group (NG, treated with 5.5 mmol/L glucose), high-glucose group (HG, treated with 30 mmol/L glucose), HG+siNC group (transfected with siNC and treated with 30 mmol/L glucose), and HG+siLUCAT1 group (transfected with siLUCAT1 and treated with 30 mmol/L glucose). (b) Left: microphotographs of cells in the Transwell assay; right: a bar graph of the average number of migrated cells. (c) Left: a scanned image of the western blot; right: a bar graph of relative protein levels. The relative protein levels were calculated according to the integrated optical density (IOD) of each band on the X-ray film using the following formula: relative protein level = IOD of the target protein/IOD of GAPDH.

Having established that LUCAT1 expression was upregulated in HG-treated HK-2 cells, three siRNAs targeting the lncRNA were designed to knock down its expression in the cells. As shown in Figure 2(a), siLUCAT1-3 exerted the most interference and thus was chosen for the subsequent assays under the label siLUCAT1.

Next, we analyzed the effects of LUCAT1 knockdown on the migration capability of HG-treated HK-2 cells and on their expression of EMT-related proteins. As revealed by the Transwell assay, the number of migrated cells was significantly higher in the HG group than in the NG group (Figure 2(b)). Moreover, HG treatment resulted in a decreased level of E-cadherin and increased levels of vimentin, alpha-smooth muscle actin (*α*-SMA), and fibronectin (FN) relative to the levels in the NG-treated cells (Figure 2(c)). To investigate whether LUCAT1 knockdown affects the EMT of HG-treated HK-2 cells, the cells were transfected with siNC or siLUCAT1 and cultured under a HG condition. Compared with the number of cells and respective levels in the HG+siNC group, the number of cells that migrated in the HG+siLUCAT1 group decreased, E-cadherin level increased, and vimentin, *α*-SMA, and FN levels decreased.

### 3.3. LUCAT1 Knockdown Inhibits the Phosphorylation of SMAD3

**Figure 3:**
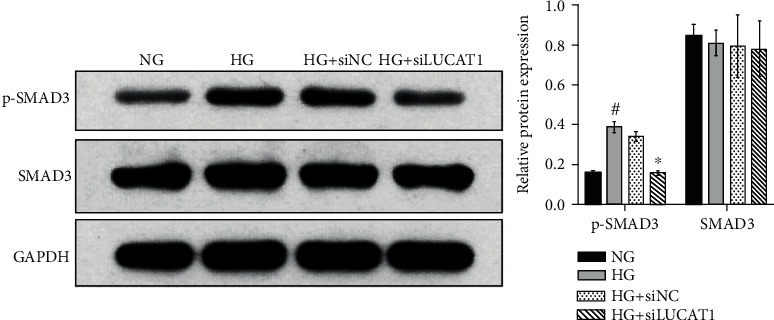
Knockdown of LUCAT1 inhibits the phosphorylation of SMAD3. HK-2 cells were divided into the normal glucose group (NG, treated with 5.5 mmol/L glucose), high-glucose group (HG, treated with 30 mmol/L glucose), HG+siNC group (transfected with siNC and treated with 30 mmol/L glucose), and HG+siLUCAT1 group (transfected with siLUCAT1 and treated with 30 mmol/L glucose). Left: a scanned image of the western blot; right: a bar graph of relative protein levels. The relative protein level was calculated according to the integrated optical density (IOD) of each band on the X-ray film using the following formula: relative protein level = IOD of the target protein/IOD of GAPDH.

To explore the underlying mechanism of LUCAT1 knockdown, we investigated its effect on the phosphorylation of SMAD family member 3 (SMAD3) using western blotting. As shown in Figure 3, p-SMAD3 level was higher in the HG group than that in the NG group, whereas it was lower in the HG+siLUCAT1 group than that in the HG+siNC group. There were no marked differences in the total SMAD3 levels among the four groups.

### 3.4. The miR-199a-5p–ZEB1 Axis Is a Downstream Target of LUCAT1

**Figure 4:**
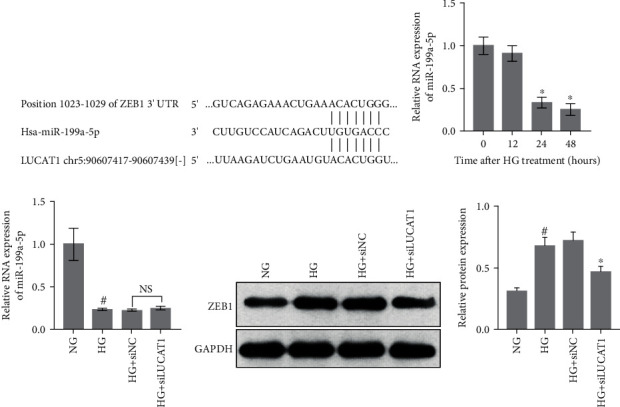
The miR-199a-5p–ZEB1 axis is a downstream target of LUCAT1. (a) Binding sites of miR-199a-5p on LUCAT1 and ZEB1 mRNA. (b) Measurement of the miR-199a-5p levels in high-glucose- (HG-) treated HK-2 cells by using quantitative reverse transcription PCR. (c, d) miR-199a-5p and ZEB1 levels in the normal glucose group (NG, treated with 5.5 mmol/L glucose), high-glucose group (HG, treated with 30 mmol/L glucose), HG+siNC group (transfected with siNC and treated with 30 mmol/L glucose), and HG+siLUCAT1 group (transfected with siLUCAT1 and treated with 30 mmol/L glucose). (d) Left: a scanned image of the western blot; right: a bar graph of the relative protein levels. The relative protein level was calculated according to the integrated optical density (IOD) of each band on the X-ray film using the following formula: relative protein level = IOD of ZEB1/IOD of GAPDH.

lncRNAs can act as competing endogenous RNAs (ceRNAs), which bind to microRNAs through microRNA response elements (MREs) and exert effects on the gene silencing caused by a specific microRNA. Using an online software prediction tool, we found that both LUCAT1 and zinc finger E-box-binding homeobox 1 (ZEB1) have the same MREs found in miR-199a-5p (Figure 4(a)). As ZEB1 is a vital regulator of EMT, we hypothesized that LUCAT1 regulates EMT via the miR-199a-5p–ZEB1 axis. Our results indicated that miR-199a-5p level gradually decreased over 24 and 48 h of HG treatment, suggesting that this microRNA mimic may play a role in regulating the damage caused by HG conditions (Figure 4(b)). However, LUCAT1 knockdown exerted no effect on miR-199a-5p level (Figure 4(c)). Additionally, the ZEB1 level was higher in the HG group than that in the NG group, whereas it was lower in the HG+siLUCAT1 group than that in the HG+siNC group (Figure 4(d)).

### 3.5. miR-199a-5p Overexpression Inhibits Both the EMT and SMAD3 Phosphorylation in HG-Treated HK-2 Cells

**Figure 5:**
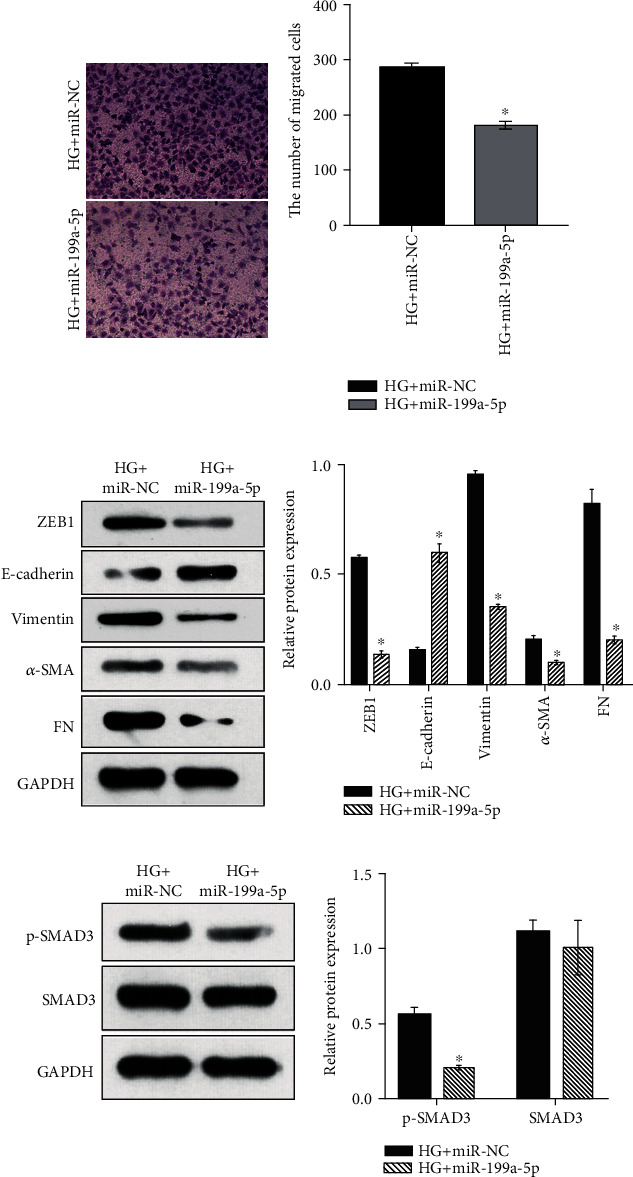
miR-199a-5p overexpression inhibits both the epithelial-mesenchymal transition (EMT) and the phosphorylation of SMAD3 in high-glucose- (HG-) treated HK-2 cells. Migration capability (a), expression level of EMT-related proteins (b), and level of phosphorylated SMAD3 (c) of cells in the HG+miR-NC group (transfected with miR-NC and treated with 30 mmol/L glucose) and HG+miR-199a-5p group (transfected with miR-199a-5p mimic and treated with 30 mmol/L glucose). (a) Left: microphotographs of the cells in the Transwell assay; right: a bar graph of the average number of migrated cells. In panels (b) and (c), the scanned images of the western blots are on the left, and the bar graphs of relative protein levels are on the right. The relative protein level was calculated according to the integrated optical density (IOD) of each band on the X-ray film using the following formula: relative protein level = IOD of the target protein/IOD of GAPDH.

To further define the role of LUCAT1 in regulating EMT via the miR-199a-5p–ZEB1 axis, we analyzed the effect of miR-199a-5p overexpression on the migration capability of HG-treated HK-2 cells and on their expression of EMT-related proteins. HK-2 cells were transfected with either miR-NC or miR-199a-5p and subsequently cultured under a HG condition. As revealed by the Transwell assay, the number of cells that migrated was significantly lower in the HG+miR-199a-5p group than that in the HG+miR-NC group (Figure 5(a)). Furthermore, compared with the corresponding levels in the HG+miR-NC group, the E-cadherin levels were increased, whereas the ZEB1, vimentin, *α*-SMA, and FN levels were decreased in the HG+miR-199a-5p group (Figure 5(b)). Moreover, p-SMAD3 level was high in the HG+miR-199a-5p group (Figure 5(c)). There was no obvious difference in the total SMAD3 levels between the two groups (Figure 5(c)).

## 4. Discussion

LUCAT1, also known as smoke- and cancer-associated lncRNA-1 (SCAL1), was first identified in the airway epithelium of cigarette smokers [15, 17]. In human bronchial epithelial cells, LUCAT1 knockdown significantly enhances the cytotoxicity induced by a cigarette smoke extract [17]. Moreover, the role of LUCAT1 has been reported to be active mainly in cancer cells, particularly, with regard to its involvement in the regulation of cancer cell migration and invasion [12–14]. Given that EMT can enhance the migration and invasion capabilities of cancer cells, we speculated that LUCAT1 may be involved in the EMT. Moreover, as HG conditions reportedly elevate LUCAT1 expression [15, 16], we hypothesized that this lncRNA may play a role in the EMT of renal tubular epithelial cells under HG conditions.

In this study, we selected HK-2 cells as the cell model for our investigation. The HK-2 cell line is an immortalized proximal tubule epithelial cell line formed from a normal adult human kidney [18]. These cells retain the functional characteristics of the proximal tubular epithelium [18] and are commonly used as a cell model in studying the tubular epithelium *in vitro*. In this study, we found that HG treatment increases LUCAT1 levels in HK-2 cells; this effect augments the possibility that this lncRNA plays a role in HG-induced injury.

The EMT of renal tubular epithelial cells accelerates the progression of renal fibrosis and is an important mechanism in the progression of diabetic nephropathy [3, 19]. Therefore, we further investigated the role of LUCAT1 in mediating the HG-induced EMT of HK-2 cells by assessing changes in the cell morphology and levels of EMT-related proteins. When renal tubular epithelial cells undergo EMT, the protein levels of epithelial markers are downregulated while those of mesenchymal markers are upregulated [20]. Epithelial cells are tightly connected in a cobblestone-shaped morphology, whereas mesenchymal cells are spindle-shaped with increased surface protrusions. We found that HG treatment increases the protein levels of the mesenchymal markers FN, vimentin, and *α*-SMA, decreases the protein level of the epithelial marker E-cadherin, and induces morphological changes in mesenchymal cells. These results indicated that HG conditions induce EMT and that our cell model construction was successful. Subsequently, we found that LUCAT1 knockdown nullified the effects of HG conditions on the HK-2 cell morphology and EMT-related protein levels. Additionally, the knockdown of this lncRNA inhibited the HG-induced phosphorylation of SMAD3. Several researchers have already suggested that the inhibition of SMAD3 phosphorylation may prevent HG-induced EMT [21, 22]. Our results corroborated those studies, suggesting that the knockdown of LUCAT1 inhibited the EMT of HG-treated HK-2 cells.

One possible mechanism through which lncRNAs execute their roles is to function as ceRNAs. According to the ceRNA regulatory mechanism, there are a variety of MREs in mRNAs and lncRNAs. When an lncRNA and an mRNA have the same MREs, the lncRNA can act as a sponge by directly binding to the miRNA molecules to decrease the number that can be bound by the corresponding mRNAs. Thus, through the MRE bridge, lncRNAs indirectly regulate the level of mRNA expression, thereby regulating cell function. Previous studies have shown that LUCAT1 and miR-199a-5p share the same MREs and that the lncRNA can thus competitively sponge miR-199a-5p [23, 24]. All our results indicated that the miR-199a-5p–ZEB1 axis was a downstream target of LUCAT1 and that miR-199a-5p overexpression inhibited the EMT of HG-treated HK-2 cells. ZEB1 is one of the key transcription factors of EMT and plays an important role in promoting the occurrence of this process [25]. Therefore, it is reasonable to hypothesize that LUCAT1 promotes HG-induced EMT through ZEB1 by sponging miR-199a-5p.

We found that miR-199a-5p overexpression resulted in reduced SMAD3 phosphorylation level; however, there is no study that reports ZEB1 can phosphorylate SMAD3. Therefore, we speculate that miR-199a-5p regulates SMAD3 signaling through other mechanisms but not through ZEB1. We will be exploring its potential mechanisms in future studies.

In conclusion, HG treatment upregulated the expression of LUCAT1 in HK-2 cells, and LUCAT1 knockdown inhibited the EMT of the HG-treated cells. The mechanism underlying this phenomenon may be that LUCAT1 promotes HG-induced EMT through ZEB1 by sponging miR-199a-5p. This is the first study to provide a preliminary explanation for the role and molecular mechanism of LUCAT1 in HG-induced EMT in renal tubular epithelial cells. Owing to the important role of EMT in diabetic nephropathy [3, 19], we hypothesized that LUCAT1 may play a role in diabetic nephropathy. However, we did not explore the connection between LUCAT1 and diabetic nephropathy. In further studies, we will analyze the function of LUCAT1 in an animal model of diabetic nephropathy and LUCAT1 expression profile in the clinical specimens of patients with diabetic nephropathy.

## Data Availability

The datasets used and/or analyzed during the current study are available from the corresponding author.
